# Electroencephalographic Correlate of Mexican Spanish Emotional Speech Processing in Autism Spectrum Disorder: To a Social Story and Robot-Based Intervention

**DOI:** 10.3389/fnhum.2021.626146

**Published:** 2021-02-26

**Authors:** Mathilde Marie Duville, Luz Maria Alonso-Valerdi, David I. Ibarra-Zarate

**Affiliations:** Neuroengineering and Neuroacoustics Research Group, Tecnologico de Monterrey, Escuela de Ingeniería y Ciencias, Monterrey, Mexico

**Keywords:** Autism Spectrum Disorders, electroencephalography, social robot, affective prosody, emotions, emotional speech database

## Abstract

Socio-emotional impairments are key symptoms of Autism Spectrum Disorders. This work proposes to analyze the neuronal activity related to the discrimination of emotional prosodies in autistic children (aged 9 to 11-year-old) as follows. Firstly, a database for single words uttered in Mexican Spanish by males, females, and children will be created. Then, optimal acoustic features for emotion characterization will be extracted, followed of a cubic kernel function Support Vector Machine (SVM) in order to validate the speech corpus. As a result, human-specific acoustic properties of emotional voice signals will be identified. Secondly, those identified acoustic properties will be modified to synthesize the recorded human emotional voices. Thirdly, both human and synthesized utterances will be used to study the electroencephalographic correlate of affective prosody processing in typically developed and autistic children. Finally, and on the basis of the outcomes, synthesized voice-enhanced environments will be created to develop an intervention based on social-robot and Social Story^TM^ for autistic children to improve affective prosodies discrimination. This protocol has been registered at BioMed Central under the following number: ISRCTN18117434.

## Introduction

### Autism Spectrum Disorders

Diagnostic criteria for Autism Spectrum Disorders (ASD) include impairments in communication and social interactions, as well as poor abilities of emotional reciprocity and emotional states recognition (American Psychiatric Association, 2013). North American prevalence of ASD lies between 8.7/1,000 registered in Mexico between 2011 and 2012 among 8-year-old children and 18.5/1,000 registered in the United States in 2016 (Fombonne et al., 2016; Chiarotti and Venerosi, 2020). Although a wide variability in prevalence estimations due to methodological concerns has been observed, there is no doubt of a general trend to increased diagnostics of autism over the last 20 years (Chiarotti and Venerosi, 2020). The growing autistic population strengthens the necessity to better understand this condition in order to improve therapies efficacy.

Reduced amplitude and shorter latency of the mismatch negativity (MMN) component in autistic children and adults whilst listening to affective prosody stimuli has been identified as an electroencephalographic (EEG) correlate due to abnormal early pre-attentive selection cognitive processes (Lindström et al., 2016; Charpentier et al., 2018). Recent studies lead to a common agreement on the general impairment of the so-called “social brain” neural network in ASD (Sato et al., 2017; Schelinski et al., 2017). Brain areas of this network are responsible for social cognition and overlap with the Default Mode Network (Subbaraju et al., 2018) and the Action Observation Network (Delbruck et al., 2019). Those brain regions interactively work to allow the successful development of social and emotional complex skills such as social imitation, face recognition, understanding of others’ mental states, communication via verbal utterances and non-verbal behaviors, and recognition and expression of emotions. Brain areas of this networks’ interplay generally depicted as impaired in ASD mainly include the inferior frontal gyrus, the medial and dorsomedial prefrontal cortices, the amygdala, temporal areas including the temporoparietal junction, the middle temporal gyrus and the superior temporal gyrus and sulcus, the orbitofrontal cortex, and the posterior cingulate cortex (Sato et al., 2017; Subbaraju et al., 2018; Delbruck et al., 2019; Francis et al., 2019). Interestingly, the superior temporal sulcus contains “non-primary” auditory neurons involved in extracting complex acoustic information after receiving inputs from the primary auditory cortex, mainly the Heschl’s gyrus responsible for low-level acoustic feature extraction (Samson et al., 2011). The superior temporal sulcus has been characterized as the human “voice area,” responsible for voice identity recognition (Schelinski et al., 2017) and is highly involved in emotion prosody processing (Rosenblau et al., 2016). This area showed weaker activity in autistics and less functional connectivity with the amygdala compared to typically developed (TD) individuals while processing emotional prosody (Rosenblau et al., 2016). Thus, autistics may be impaired in extracting complex auditory features that characterize emotional prosody stimuli and fail to allocate social emotional value.

### Psychoacoustic Properties of Auditory Information

Low interest for speech and atypical distressing reactions to some noises (Schauder and Bennetto, 2016) usually comes along with high interest for music and intact low level auditory abilities (e.g., non-vocal pitch discrimination) in ASD (Globerson et al., 2015; Schelinski and von Kriegstein, 2019). Autistic individuals also show typical abilities to recognize emotional and pragmatic intonations in music (Quintin et al., 2011; DePriest et al., 2017). Vocal emotion recognition abilities have been associated with vocal pitch discrimination skills, and lower emotional prosody recognition capacities have been associated with higher autistic severity (Schelinski and von Kriegstein, 2019). Impairments in emotional prosody recognition observed in ASD may be triggered by psychoacoustic properties of auditory stimuli. While intonation discrimination in music and non-vocal auditory stimuli stem from the accurate processing of local information (e.g., frequency fluctuations and harmonic contour), speech is a highly noisy signal and prosody recognition requires the coordination between a coarsier local processing and higher-level ones (e.g., social allocation, complex acoustic patterns processing) (Zatorre and Baum, 2012; DePriest et al., 2017). In essence, the complex property of vocal sounds defined by its naturalness (i.e., speech comes from the human vocal tract) triggers the difficulty for autistics to discriminate emotional prosodies. In fact, the autistic processing style characterized by the highly accurate local information extraction [Enhanced Perceptual Functioning theory (Mottron et al., 2006)], along with the low ability to integrate the information into a global representation [Weak Central Coherence theory (Happé and Frith, 2006)], may be disadvantageous for developing speech processing abilities. Therefore, the present study aims to identify the physical acoustic properties that characterize naturalness (i.e., specific complexity of vocal sounds) in speech in order to modulate psychoacoustic features of voice that could improve the emotional prosody recognition in ASD.

### Emotional Prosody Recognition

Vocal emotion recognition relies at least partly on the perception of the acoustic properties of the stimulus. In fact, similar acoustic indicators (e.g., pitch, intensity) of emotional prosody have been stressed across cultures (Paulmann and Uskul, 2014), highlighting a universal reliance on the physical acoustic characteristics to recognize emotions in voice. Acoustic physical properties generally recognized as differentiators of emotional prosodies are spectral features, intensity/energy, and rate (Kamińska, 2019). In emotion classification studies, the most considered spectral features are the Mel Frequency Spectral Coefficients (MFCC) (Caballero-Morales, 2013), pitch (fundamental frequency), formants, jitter, shimmer, and harmonics-to-noise ratio (Arruti et al., 2014). So far, culture-specific ways of uttering emotions have been revealed (Paulmann and Uskul, 2014). For instance, English-speakers from Singapore tend to allocate more intensity and higher pitch when expressing anger than English-speakers from Kenya (Laukka et al., 2016). Therefore, although emotional prosody recognition relies on universal norms, cross-culture variations make each ethnic group unique in its way of uttering emotions and listeners from one ethnic group skilled at recognizing emotions uttered by speakers of their own culture. The European Union funded the Interface project to create a public database of emotional utterances in French, Castilian Spanish, Slovenian, and English languages (Hozjan et al., 2002; Emotional Speech Synthesis Database, 2012). Particularly, the Castilian Spanish database contains isolated words uttered by adult male and female voices with neutral, angry, sad, joyful, afraid, disgusted, and surprised prosodies (Hozjan et al., 2002). The Interface database was generated for research purposes to analyze and classify emotional speech. Its subjective evaluation (i.e. the emotion recognition by 16 non-professional engineering students listeners) has shown up to 90% accuracy for correct emotions recognition (Hozjan et al., 2002). Up to now, no emotional speech database in Mexican Spanish containing isolated words uttered with anger, sadness, happiness, fear, disgust, and neutral prosodies is publicly available yet (Caballero-Morales, 2013). Therefore, one aim of the present study pursues to generate a database for adult male, female, and childish voices.

### EEG Signature of Emotion Recognition

To record the brain signature of emotions differentiation in voice, soundless methods may be more suitable since the recording process does not interfere with the acoustic stimulus that has to be processed. EEG, and particularly the analysis of Event-Related Potentials (ERPs), has been widely used to study the neuronal activation process of emotions. Furthermore, thanks to its high temporal resolution, ERPs analysis enable the distinction within attentional, sensory, and higher cognitive stages (Fan and Cheng, 2014). Early automatic auditory perception is usually observed between 100 and 250 ms over frontal and central cortices after stimulus onset (MMN). Higher amplitude of this component indicates higher discrimination of acoustic modulations relative to constant auditory background (Charpentier et al., 2018). As an index of acoustic changes early discrimination, the MMN can be used as an indicator of emotional prosody detection (Lindström et al., 2016, 2018; Paris et al., 2018). If automatic changes discrimination was salient enough, involuntary attentional switch to deviant auditory stimuli happens, and is observed 200–350 ms after stimulus onset at fronto-central cortices as a component known as P3a (Lindström et al., 2018; Paris et al., 2018). Early emotional recognition, valence, and arousal identification appear over frontal and central electrodes between 250 and 350 ms after the stimulus onset (Paulmann et al., 2013). The analysis of the amplitude of the P200 component has allowed to distinguish different prosodies such as disgust (high amplitude) and fear (lower amplitude) (Paulmann et al., 2013). This component has shown to reflect the early arousal decoding and may predict the deeper processing of the emotional information observed in the 250–1,000 ms time-window after the stimulus onset (Paulmann, 2016; Steber et al., 2020). The time window between 400 and 1,000 ms has been related to the motivational relevance and extensive processing of the prosodic emotional stimulus. In such window, the observation of the Late Positive Potential (LPP) over centro-parietal recording sites is frequent (Steber et al., 2020). The LPP amplitude is higher in emotional than neutral stimuli, while it is shorter during the emotional down-regulation (Pan et al., 2020).

Event-Related Potentials laterality is still a controversy. Some experimental paradigms have found a right-hemispheric dominance for emotional processing, regardless of the valence (result in accordance with the Right-Hemispheric Hypothesis) (Godfrey and Grimshaw, 2016; Charpentier et al., 2018). The left lateralization for the processing of positive emotions and higher right hemispheric neuronal activity for negative emotions (i.e., the Valence Theory) has also been observed, without denying the possibility of the mutual coexistence of both phenomena (Wyczesany et al., 2018). Although a sparse literature relative to the ERPs analysis underlying the processing of emotional prosody in autistics, a reduced modulation of the neuronal activity by emotions and a lack of hemispheric lateralization has been observed in this population (Lindström et al., 2016, 2018; Charpentier et al., 2018). Remarkably, in a study focused on the processing of emotional words by autistic adults and their corresponding TD controls, the amplitude of the LPP for positive or negative versus neutral words processing did not reach significance for autistic participants. In contrast, higher amplitude were observed for emotional versus neutral stimuli in the TD sample, and group differences were identified for negative versus neutral words (Lartseva et al., 2015).

### Assistive Technology for the Improvement of Emotional Skills in ASD

#### Social Stories^TM^

Various educational approaches have been proposed to improve social and emotional skills in autistics. For example, the so-called Social Story^TM^ intervention drawn-up by Carol Gray showed promising results (Gray, 2010; Kurt and Kutlu, 2019). This practice consists of sharing relevant cues via a rigorous scheme to teach the patient to process correctly specific every-day life situations (Kurt and Kutlu, 2019). Gray (2010) suggested the following advices. First, the content of the stories should be highly personalized to each patient in order to guarantee the comprehension and attention of the patient. Second, third persons and generalizations may help to keep self-esteem safe (Gray, 2010). Third, format, number of sentences, rimes, rhythm must be adapted to the age, preferences, and cognitive skills of the audience (Bee et al., 2018). Fourth, the use of descriptive, perspective, coaching and affirmative sentences would help to convey accurate information (Bee et al., 2018). Finally, interactions with the patient via questions and partial sentences help to maintain the audience focused, which facilitates to monitor the progression of the patient (Gray, 2010). Overall, the intervention must be implemented in a safe, pleasant and friendly environment with the general aim of fostering the learning (Gray, 2010).

Interventions by Social Stories^TM^ present several advantages: thanks to its easy implementation, parents, caregivers, or close friends can help to personalize the stories, and their content can be updated at wish. On the other hand, improvements can be observed in brief implementations and short sessions (5-min sessions over a 1 to 4-week period) (Vandermeer et al., 2015; Kurt and Kutlu, 2019). The Social Story^TM^ approach has been recently adapted to technology-based interventions to enhance the motivation of patients, facility and efficacy of the interventions (Miller et al., 2020).

#### Social Robots

New technologies help to create a safe and predictable social environment, in which the complexity of social cues can be progressively controlled through the intervention time course, thereby generating an optimized atmosphere for autistic patients (Sartorato et al., 2017). In this respect, social robots offer direct, real, and multisensorial social interactions that have been shown to be perceived as “natural” as human ones by autistics (Chaminade et al., 2012). Social robot-based interventions have shown to improve emotion interpretation differentiation, and emotional perspective-taking skills in autistic children. Eventually, those improvements could be generalized to human and daily life social interactions (Scassellati et al., 2018; Marino et al., 2019). As social robots are accepted by autistics and equally perceived as social partners as humans, they give the opportunity to train social skills.

### Current Study

In the light of research advances, the present proposal aims to integrate Social Stories^TM^ and social robots to improve emotional prosody recognition in Mexican autistic children population. This research will be conducted in three stages:

(1) *Mexican Emotional Speech Database (MESD)*: Characterization of emotional prosodies by physical acoustic features in Castilian and Mexican Spanish cultures. Cultural differences will be identified and an emotional speech database for Mexican Spanish will be generated.
(2) *Human vs Synthesized Voice*: Defining the naturalness in human speech by physical acoustic properties. Thereafter, previously recorded emotional Mexican Spanish utterances will be edited in order to reduce naturalness in voice. Finally, the neuronal processing of human and newly synthesized emotional utterances will be recorded by EEG and compared between TD and autistic children.
(3) *Social Story*^TM^ *and Robot Intervention*: The voices that lead to the better approximation to a typical processing of emotions in stage 2 will be used to implement a Social Story^TM^ and Robot based intervention. The integrative intervention will be tested on a sample of 54 autistic children divided in 1 experimental group of 18 children and 2 control group composed of 18 children each, and 18 TD children, between 9 and 11 years.

## Materials and Methods

### Mexican Emotional Speech Database

First, it is necessary to characterize physical acoustic features of voice recordings uttered with neutral and emotional prosodies for Mexican Spanish language. The main objective of this stage is to create a Mexican Emotional Speech Database (MESD) encompassing adult male, female, and child voices.

#### Sample

Four healthy male, four healthy female adults (between 19 and 35 years), and eight healthy children (between 9 and 11 years) (Stathopoulos et al., 2011) will voluntary participate to speech recordings. Participants will be excluded if they have been diagnosed with any pathology that affects emotional behavior, hearing, or speech, or present sickness traits affecting voice timbre at the time of the study. Participants will be included if they have grown up in Mexico in a cultural Mexican environment (Mexican academic education and family environments) in order to guarantee a Mexican-way of conveying emotions. Participants will be recruited from a public announcement posted on Tecnologico de Monterrey, Campus Monterrey.

#### MESD Speech Corpus Stimuli

Words for speech utterances will be selected from two sources: the single-word corpus from the INTERFACE for Castilian Spanish database (Hozjan et al., 2002; Emotional Speech Synthesis Database, 2012), and the Madrid Affective Database for Spanish (MADS; Hinojosa et al., 2016a, b). For the creation of the MESD, the 24 isolated words that composed the INTERFACE database will be selected and will repeat across emotions. Words from MADS will be selected based on grammatical class and ratings for valence, arousal, qualitative emotion (anger, disgust, fear, happiness, neutral, and sadness), concreteness, familiarity, frequency, and subjective age of acquisition. Specifically, all words will be nouns or adjectives. Their subjective age of acquisition will be strictly under 9-year-old. Valence and arousal ratings of neutral words will be strictly greater than 4 but lower than 6 (on a 9-point scale), whereas emotional words will be selected to have valence and arousal ratings ranging from 1 to 4, or from 6 to 9. A rating superior to 2.5 (on a 5-point scale) for a particular emotion will allow the qualitative categorization of the word into anger, disgust, fear, happiness, or sadness. Words from MADS will be selected in order to ensure that they will be matched for concreteness, familiarity, and frequency ratings. Words selection will be carried out separately for male, female, and mean ratings for all subjects.

Statistical analysis will be used to guarantee that frequency, familiarity, and concreteness are matched between neutral, fear, disgust, happiness, sadness, and anger words. Values obtained for each of these parameters will be analyzed separately with one-way ANOVAs. Normality and homogeneity will be assessed using Shapiro–Wilk and Bartlett tests, respectively. In case of non-parametric distribution of the data, Kruskal–Wallis test will be applied. *Post hoc* tests will be used to statistically assess specific differences (Tukey after ANOVA, Wilcoxon tests with *p*-value adjustment by Holm method after Kruskal–Wallis). In case of significant differences between emotions, outlier values will be identified, and corresponding words will be eliminated until reaching no difference for all of three parameters. We will consider outliers all ratings for frequency, familiarity or concreteness outside the range defined by percentiles 2.5 and 97.5.

Sample size estimation was calculated with G^∗^Power software version 3.1.9.7 (Faul et al., 2007) for Cohen’s d effect size index of 0.8, statistical significance set at *p* < 0.05. Results indicated that 19 words in each emotion would be enough to provide an estimated power of 0.92. The total speech corpus will be composed of 72 words per emotion (24 from INTERFACE and 48 from MADS).

As ratings might vary from Spanish to Mexican cultures (Hinojosa et al., 2016a, b), cross-culture validation will proceed as follows. Two questionnaires will be created using Survey Monkey online Software. On one questionnaire, participants will have to rate familiarity and concreteness; and on the other hand, they will rate valence, arousal, and qualitative emotion (anger, disgust, fear, happiness, neutral, or sadness). The 288 words selected from MADS will be randomly assigned to four lists of 72 words for estimated 20–25 min questionnaires. All lists will be assigned to both questionnaires. Questionnaires will start with participants required to answer demographic questions about their age and gender. Participants will be able to add an e-mail address if they want to enquire about the research. Then, questionnaire purposes and instructions (information about the rating of each variable) will be explained. Instructions will be adapted from the one proposed by Hinojosa et al. (2016a, b) and are detailed in Table 1. Frequency of use will be taken from LEXMEX (Silva-Pereyra et al., 2014).

**TABLE 1:** Instructions for participants to rate words variables.

Variable	Instructions
Familiarity	It refers to how frequently you encounter the word in your day-to-day language, including both written and oral forms. Please rate familiarity in a 9-point scale, 1 being that you very sparsely find the word in your ordinary language, and 9 being that the word is very frequent in your ordinary language
Concreteness	It refers to how concrete versus abstract you think a concept is. For instance, “device” is an abstract concept because it can refer to a wide set of objects, whereas “foot” is a concrete word because it refers to only one specific concept. Please rate concreteness using a 9-point scale: 1 being “very abstract,” 9 being “very concrete”
Valence	It refers to how much you consider a concept negative/aversive versus positive/attractive. Please rate valence using a 9-point scale: 1 being “very negative,” 9 being “very positive”
Arousal	It refers to the level of relaxation versus excitation a concept generates. Please rate arousal using a 9-point scale: 1 being “very low arousal (very relaxing/calming),” 9 being “very high arousal (very stimulating)”
Qualitative emotion	Please rate the word according to how its concept could be associated with the emotions: anger, disgust, fear, happiness, and sadness. Use a 5-point scale, 1 being “nothing at all,” and 5 being “extremely”

Each word will be presented alone in a page, written in 18-point Arial font. The variables to rate with their respective scale will be placed below the word, so that the participant can answer. Scales will be accompanied with labels of extreme values to allow the participant retaining the direction of the polarity of the dimensions. Familiarity will be the first variable to rate, so that participants will not be biased from rating other variables for the same word first. All items will include the optional answer, “I do not know the meaning.” Each question will be able to be answered once. The order of word apparition will be randomized for each participant.

Mean and standard deviation scores for male, female, children, and all together ratings will be calculated for each word. For culture comparisons, one-way ANOVAs will be conducted for each emotion and variable separately on words qualified as referring to a specific emotion in both cultures. Sample size estimation was calculated with G^∗^Power software version 3.1.9.7 (Faul et al., 2007) for Cohen’s d effect size index of 0.8, and statistical significance set at *p* < 0.05. Results indicated that 34 participants in each category (adult male, adult female, and child) would be enough to provide an estimated power of 0.90. For emotion comparisons in Mexican ratings, values obtained for each variable will be analyzed separately with one-way ANOVAs. Normality and homogeneity will be assessed using Shapiro–Wilk and Bartlett tests, respectively. In case of non-parametric distribution of the data, Kruskal–Wallis test will be applied. *Post hoc* tests will be used to statistically assess specific differences (Tukey after ANOVA, Wilcoxon tests with *p*-value adjustment by Holm method after Kruskal–Wallis). In case of cross-cultural differences, outlier values from both Spanish and Mexican ratings will be identified, and corresponding words will be eliminated until reaching no cultural difference, and no emotional difference for Mexican ratings for all of three parameters (familiarity, frequency, and concreteness). We will consider outliers all ratings for frequency, familiarity or concreteness outside the range defined by percentiles 2.5 and 97.5. The final speech corpus from MADS will be composed of 144 words (24 per emotion), so that the final MESD corpus will be composed of 48 words per emotion (24 from INTERFACE and 24 from MADS).

Forty Mexican participants of each category (adult male, adult female, and child) will voluntarily answer the questionnaires. Adults will be aged between 19 and 35 years old, and children between 9 and 11 years old. They will be excluded if they have been diagnosed with any pathology that affects emotional behavior. They will be included if that have grown up in Mexico in a cultural Mexican environment (Mexican academic education and family environments). Participants will be recruited via public posted on Tecnologico de Monterrey, Campus Monterrey.

In all cases, significance will be set at *p* < 0.05. Statistical analysis will be performed with R software (R Foundation for Statistical Computing, Vienna, Austria).

#### Speech Recordings Elaboration and Procedures

Recordings will be carried out in a professional recording studio using the following material: (1) a Sennheiser e835 microphone, (2) a Focusrite Scarlett 2i4 audio interface connected to the microphone with an XLR cable and to the computer, and (3) the digital audio workstation REAPER (Rapid Environment for Audio Production, Engineering, and Recording) will be used to generate and record audio files that will be stored as a sequence of 24-bit with a sample rate 297 of 48000 Hz.

Sessions will last approximately 1 h for adults and 30 min for children. Adults will be asked to utter 288 words, that is 48 words per emotion, being 24 coming from INTERFACE, and 24 coming from MADS. Four children will utter the selected words from MADS (for a total of 144 words), and four children will express the words coming from INTERFACE (144 utterances). The order of word corpora will be counterbalanced across adult sessions, and emotions will be randomly distributed for both cases: adults and children. Participants will be asked to read and get grips with the entire word dataset and will be given as much time as they require. Then, they will be instructed to read each 24-word list with one of six emotional intonations: neutral, happy, angry, sad, disgusted, or afraid. Children who will express words coming from MADS will be presented with the corpora of words selected based on mean ratings for all subject. Adults male and female will utter words based on selection from mean ratings for males and females, respectively. Participants will be asked to do their best possible to get into the emotional role. All words from a particular emotion will be uttered successively to facilitate this process. Participants will be asked to wait at least 5 s between two utterances in order to focus before each recording.

#### Sound Processing and Analyses

Each word will be excerpted from the continuous recording of each session to generate individual word audio files. Then, Praat and Matlab R2019b will be used to extract acoustic features as follows (Swain et al., 2018).

Prosodic features:

- Fundamental frequency, or pitch (Hz): mean and standard deviation over the entire waveform.
- Speech rate: number of syllables per second.
- Root mean square energy (Volts): square root of mean energy.
- Intensity (dB): mean and standard deviation over the entire waveform.

Voice quality features:

- Jitter (%): it is an index of the periodic fluctuation of fundamental frequency. Average absolute difference between two consecutive periods, divided by the average period (jitter local). Average absolute difference between a period and the average of it and its four closest neighbors, divided by the average period (jitter ppq5).
- Shimmer (%): it is an index of the periodic fluctuation of the signal amplitude. Average absolute difference between the amplitude of two consecutive periods, divided by the average amplitude (shimmer local). Average absolute difference between the amplitude of a period and its mean amplitude and its four closest neighbors, divided by the average amplitude (shimmer ppq5).
- Mean harmonic-to-noise ratio (dB): mean ratio of the energy of the harmonics to the energy of the remaining part of the signal. It will be calculated as described by Eqs 1 and 2:

If 99% of the signal is composed of harmonics and 1% is noise, then:

(1) $$\text{Harmonic-to-noise ratio}=10\log_{10}\left(\frac{99}{1}\right)=20\text{dB}$$

Mean HNR between time point t1 and time point t2 is defined as:

(2) $$\text{Mean harmonic-to-noise ratio}=\frac{1}{\left(t_{2}-t_{1}\right)}\int_{t_{1}}^{t_{2}}dtx\left(t\right)$$

where *x*(*t*) is the harmonic-to-noise ratio (in dB) as a function of time.

Spectral features:

- Mean first, second and third formants (Hz): mean and bandwidth in center.
- Mel Frequency Cepstral Coefficients: 1–13.

In order to reduce inter-individual biases due to physical anatomy (e.g., body corpulence), values from each parameter extracted will be rescaled between 0 and 1 according to the max-min normalization. The formula expressed in Eq. 3 will be used:

(3) $$x_{\text{normalized}}=\frac{x-\min_{kj}}{\max_{kj}-\min_{kj}}$$

Where *x* is the value to be normalized, *max*_*kj*_ is the highest value of acoustic feature *k* and *min*_*kj*_ is the lowest value of *k*.

For emotion comparisons, a one-way ANOVA will be applied on each acoustic feature and type of voice separately, excepting MFCC coefficients. Normality and homogeneity will be assessed using Shapiro–Wilk and Bartlett tests, respectively. In case of non-parametric distribution of the data, Kruskal–Wallis test will be applied. *Post hoc* tests will be used to statistically assess specific differences (Tukey after ANOVA, Wilcoxon tests with *p*-value adjustment by Holm method after Kruskal–Wallis).

To evaluate the effect of controlling frequency, familiarity, and concreteness, a Corpus (words selected from MADS versus words selected from INTERFACE) × Emotions ANOVA will be conducted on each acoustic features separately. Normality and homogeneity will be assessed using Shapiro–Wilk and Bartlett tests, respectively. In case of non-parametric distribution of the data, a transformation will be performed. *Post hoc* comparisons will be conducted to assess specific differences (Tukey).

For culture comparisons, previously mentioned features will be extracted also from INTERFACE database audio files for (1) Anger, (2) Disgust, (3) Fear, (4) Joy, (5) Neutral/normal, and (6) Sadness. Only the data from the INTERFACE word corpus found in the MESD will be considered to allow comparisons with the Castilian Spanish dataset. A repeated measures Cultures (Spanish vs Mexican) × Emotions ANOVA will be performed for each acoustic feature separately. Normality will be assessed by Shapiro–Wilk test. In case of non-parametric distribution of the data, a transformation will be applied. Mauchly’s test of sphericity will be used to evaluate homogeneity of variances and co-variances. In case of violation of sphericity, a Greenhouse-Geisser correction will be applied in order to test main effects significance. *Post hoc* comparisons will be conducted to assess specific differences (Tukey).

For all analysis, level of significance will be set at *p* < 0.05. Statistical analysis will be performed with R software.

#### Emotions Classification

Matlab R2019b will be used to carry out a supervised learning analysis using a SVM predictive model on acoustic features extracted from MESD and from INTERACE separately. Hyperparameters will be adjusted to a cubic kernel function and a box constraint level (soft-margin penalty) at 10. The multiclass method (one-vs-one or one-vs-all) and the kernel scale parameters will be set to “auto,” meaning that the algorithm will be automatically optimized for both parameters according to the dataset. The dataset will be divided into two groups: training and validation, corresponding to 77 and 23% of the entire dataset, respectively. A stratified train/test split holdout cross-validation method will be used, so that data training and validation will present an equal number of words per emotion. A dimensionality reduction using Principal Component Analysis, explaining 95% of the variance were applied before classification analysis (Liu et al., 2018).

The following parameters will be computed in order to evaluate the classifier performance (Tharwat, 2020):

- *Accuracy* is the ratio between number of tuples correctly classified and the total number of tuples.
- *Precision* represents the relation of the number of correctly classified positive tuples against the total number of tuples classified as positive, including true and false positives. It is an index of exactness of the predictive model.
- *Recall* represents the relation of the number of positive tuples correctly classified against the total number of positive tuples, including true positives and false negatives. It is an index of the completeness of the predictive model.
- *F-score* is the harmonic mean of precision and recall. It reflects the balance between precision and recall.

A classification analysis will be applied individually on each adult participant and male, and female recordings will analyzed separately. The final dataset of the MESD will be created by selecting for each emotion, the dataset from the participant who the best F-score will be reached. This selection will allow to approximate non-professionalism of actors biases compensation. Classification analysis will be applied on the final 288-utterance dataset of MESD.

For children datasets, a k-means clustering analysis will be applied on features extracted for each emotion separately (24 observations per participant, leading to six datasets of 192 observations). Squared Euclidean distance metric and k-means++ algorithm for cluster center initialization will be used. The optimized number of clusters will be assessed by computing silhouette scores. The number of clusters that will lead to the highest average silhouette score will be selected. In each cluster, utterances of words coming from INTERFACE word corpus (four participants) and MADS (four participants) will be considered separately. For utterances of both word corpora, the number of observations of each participant in each cluster will be computed. Pairs of participants (one who uttered words from MADS, and one who uttered word from INTERFACE) will be assessed in each cluster by considering the participant of highest number of observations for each corpus. As a result, each pair of participants will be composed of 288 utterances (48 per emotion, including 24 of words from INTERFACE word corpus and 24 of words from MADS). A classification analysis will be carried out on data from each resulting pair. The final dataset of the MESD will be created by selecting for each emotion, the dataset from the pair of participants for which the best F-score will be reached. A classification analysis will be applied on the final 288-utterance dataset of MESD.

For the MESD database to be validated, we expect to observe approximately the same classification performance for both MESD and INTERFACE database.

### Synthesized and Human Emotional Utterances Processing in Autistic Children

**FIGURE 1:**
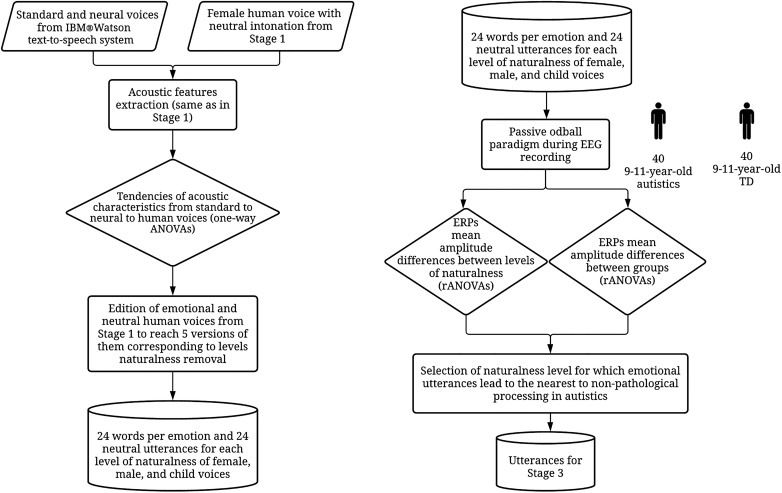
Process of Stage 2. TD, Typically developed.

This stage of the research aims to edit speech auditory files previously recorded in the first stage in order to synthesize “less natural” voices that would help to approximate a typical emotional processing in autistic children. First of all, naturalness in speech signals will be defined by analyzing standard and neural synthesized voices coming from IBM^®^ Watson text-to-speech system. Their acoustic parameters will be statistically compared to human voice recordings from Stage 1. Analyzed features will cover spectral characteristics, intensity, and duration. Then, frequencies will be filtered, intensity and total duration of speech files will be edited in order to create five types of synthesized voices that correspond to five levels of naturalness: level 1 will be the less natural voice (i.e., approximates standard synthesized voice from IBM^®^ Watson text-to-speech system) and level 5 will be the more natural voice (i.e., approximates human speech). Human and synthesized voices will be further listened and processed by autistic children and matched TD controls while behavioral responses and EEG activity will be registered. Finally, the one synthesized voice processed by autistic participants that will lead to the nearest to human speech EEG processing by controls will be selected for the third stage of the research. The process of Stage 2 is illustrated in Figure 1 and detailed as follows.

#### Standard and Neural Speech Files Acquisition

The IBM^®^ Watson text-to-speech system will be used to generate audio files for further analysis. This application proposes a neutral North-American Spanish female voice that will be used in its two versions: standard and enhanced. The former is created by a concatenative synthesis that uses previously recorded speech 10–100 ms segments which represent units that are assembled according to the input text. Discontinuities generated by the process of assemblage lead to a partial degradation of speech naturalness. On the other hand, enhanced or neural synthesized voices are created by Deep Neural Networks able to predict speech acoustic features trained on human speech. This method leads to greater level of naturalness in the output signal (see Baird et al., 2017 for more details about voices synthesis methods). At this stage, neutral words from MESD (MADS corpus) will be entered in the text-to-speech application and their standard and enhanced corresponding audio signals will be downloaded.

#### “Naturalness in Voice” Characterization

Spectral, intensity and duration features (extracted parameters will be the same than the ones extracted in stage 1) of audio signals from standard, neural and human voices will be extracted. As standard and neural synthesized voices are female and neutral, the human adult female voices with neutral prosody will be used for comparisons. A statistical analysis will be carried out to compare those voices and explore tendencies of acoustic characteristics from standard to neural to human voices. One-way ANOVAs will be conducted for voices comparisons on each extracted parameter separately. Normality and homogeneity will be assessed using Shapiro–Wilk and Bartlett tests, respectively. In case of non-parametric distribution of the data, Kruskal–Wallis test will be applied. *Post hoc* comparisons will be conducted to assess specific differences (Tukey procedure in case of ANOVA, Wilcoxon tests with *p*-value adjustment by Holm method in case of Kruskal–Wallis test). Statistical analysis will be performed with R software and level of significance set at *p* < 0.05.

Sample size estimation was calculated with G^∗^Power software version 3.1.9.7 (Faul et al., 2007) for Cohen’s d effect size index of 0.9, statistical significance set at *p* < 0.05. Results indicated that 22 words in each voice type (standard, enhanced, and human) would be enough to provide an estimated power of 0.91. As a result, 24 words per voice type will be used.

#### Creation of Five Levels of Naturalness for Emotional Speech

Once acoustic features tendencies across naturalness levels will be defined, audio files from emotional human voices recordings will be edited in order to reduce naturalness in those speech signals. Spectral content will be filtered, intensity level and total duration (speech rate) will be adjusted. Acoustic signals edition will be realized using Matlab. For instance, if less naturalness is characterized by higher levels of harmonics-to-noise ratio [human speech is a noisy and continuous signal (Zatorre and Baum, 2012)], then frequencies that are not harmonics will be filtered. The edition of the signal will be applied on previously recorded in Stage 1 audio files for adult male, female, and children voices. Anger, fear, sadness, disgust, happiness, and neutral acoustic signals will be similarly adjusted. Naturalness will be progressively reduced to generate five different levels: from level 1: the less natural to level 5: the more natural voice. Level 1 will correspond to a 95% removal of naturalness, levels 2–5 will correspond to a 76, 57, 38, and 19% diminution, respectively. In accordance with this example, if less naturalness is characterized by higher levels of harmonics-to-noise ratio, then 95% of frequencies that are not harmonics will randomly be filtered for level 1, 76, 57, 38, and 19% of non-harmonics frequencies will be filtered for levels 2–5, respectively. All features defined as being part of naturalness characterization will be edited simultaneously.

Statistical analyses will be used to ensure that levels 1–5 and human voice differ as regard the acoustic parameters edited. Values obtained for each voice and emotion (e.g., adult male/anger, adult female/happiness, etc.) will be analyzed separately with one-way ANOVAs. Normality and homogeneity will be assessed using Shapiro–Wilk and Bartlett tests, respectively. In case of non-parametric distribution of the data, Kruskal–Wallis test will be applied. *Post hoc* comparisons will be conducted to assess specific differences (Tukey after ANOVA, Wilcoxon tests with *p*-value adjustment by Holm method after Kruskal–Wallis). In case of no statistical difference between levels, percentages of diminution of naturalness will be adjusted. Levels must differ for all parameters implicated in naturalness characterization.

Sample size estimation was calculated with G^∗^Power software version 3.1.9.7 (Faul et al., 2007) for Cohen’s d effect size index of 0.9, statistical significance set at *p* < 0.05. Results indicated that 17 words in each level would be enough to provide an estimated power of 0.92.

As all emotions will be edited similarly, relations between emotions will be preserved [e.g., stage 1 would highlight that harmonics-to-noise ratio for happiness > harmonics-to-noise ratio for sadness (Scherer et al., 2011)]. Therefore, the differentiation between emotions will not be affected by editing the level of naturalness of the voice. However, statistical analyses will be applied to verify this assumption after voices synthesis. For comparisons between emotions, values obtained for acoustic features listed in stage 1 will be analyzed for each voice separately with one-way ANOVAs. Normality and homogeneity will be assessed using Shapiro-Wilk and Bartlett tests, respectively. In case of non-parametric distribution of the data, Kruskal-Wallis test will be applied. Post-hoc comparisons will be conducted to assess specific differences (Tukey after ANOVA, Wilcoxon tests with *p*-value adjustment by Holm method after Kruskal–Wallis).

Sample size estimation was calculated with G^∗^Power software version 3.1.9.7 (Faul et al., 2007) for Cohen’s d effect size index of 0.8, statistical significance set at *p* < 0.05. Results indicated that 19 words in each emotion would be enough to provide an estimated power of 0.92.

In all cases, statistical analysis will be performed with R software and level of significance will be set at *p* < 0.05.

#### Passive Oddball Paradigm

Emotional and neutral words uttered by human voices recorded in stage 1 and synthesized voices created in stage 2 will be displayed to the participant. During the EEG recording, participants will sit on an armchair while watching a silent movie and will be instructed to pay no attention to the auditive stimuli. To facilitate the comparison of naturalness levels, six stimulus blocks will be created, each containing 600 stimuli corresponding to one of the five levels, or the human voice. Each block will consist of 480 frequently presented neutral-uttered standard stimuli (80%), and occasional 24 anger (4%), 24 disgust (4%), 24 fear (4%), 24 happiness (4%), and 24 sadness (4%) deviant stimuli. Standard and deviant stimuli will be equally uttered with a male, woman, or child voice. The stimuli will be presented pseudo-randomly with deviant stimuli being preceded by at least two standard stimuli. Each participant will listen to three blocks for an approximatively 30-min task. The stimulus sequences for each block will be fixed, and the order of blocks will be counterbalanced and randomized between participants. The stimulus onset asynchrony will be of 1,300 ms. The stimuli will be presented at 60 dB via the Shure SRH1840 audio headset that has a flat response frequency response in order to accurately reproduce the input audio signal. Autistic participants will be accompanied by their parent if necessary.

#### EEG Recording and Processing

Electroencephalographic signals will be recorded with a 10/20 International System with 24 channels (Greentek Gelfree S3, mBrain Train) with a sampling rate of 500 Hz into a bandwidth between 0.1 and 100 Hz. EEG Lab toolbox from Matlab will be used to preprocess and process the data. Before data analysis, the signal will be referenced to the average between left and right mastoids. Electrode impedance will be kept below 5 kΩ. EEG data will be epoched over a window time of 1,100 ms, including a pre-stimulus 100 ms for baseline correction. A High-pass filter of 0.1 Hz and a low-pass filter of 50 Hz will be applied before epoching. The removal of eye movements and muscle artifacts will be based on the independent component analysis method using the ICLabels plug-in of EEG Lab. Epochs with a voltage exceeding 100 μV will be removed from data analysis.

#### Data Analysis

For ERP amplitude analysis, electrodes will be grouped according to regions of interest (ROIs). Left prefrontal site: FP1. Left frontal channels: F3, F7. Right prefrontal site: FP2. Midline pre-frontal site: AFz. Midline frontal electrode: Fz. Right frontal channels: F4, F8. Left temporal site: T7. Left central electrode: C3. Midline central electrode: Cz. Right temporal site: T8, Right central electrode: C4. Centro-parietal electrode: CPz. Left parietal sites: P3, P7. Midline-parietal electrode: Pz. Right parietal sites: P4, P8. Midline parieto-occipital electrode: POz. Left occipital site: O1. Right occipital site: O2. Maximum peak time window analysis will be: 100–250 ms for the MMN (Kujala et al., 2007; Paris et al., 2018), 170–230 ms for P200 component (Paulmann et al., 2013), 200 and 350 ms for the P3a (Kujala et al., 2007; Paris et al., 2018), and 400–1,000 ms for the LPP component (Steber et al., 2020).

Event-Related Potentials will be extracted by averaging the data obtained for standard and deviant stimuli, and for each condition independently. The ERPs elicited by standard stimuli will be subtracted from the ERPs elicited by deviants to calculate difference waves. The mean ERPs amplitude will be calculated as follows. For standard stimuli, level of naturalness and group average peak latencies will be determined from all ROIs. Mean amplitudes will be determined by considering a ±50-ms time window centered at the grand mean peak latency. For deviant stimuli, mean amplitudes will be calculated from the difference waves at ROIs. Mean amplitudes will be determined by considering a ±50-ms time window centered to the grand mean peak latency (Kujala et al., 2007; Paris et al., 2018).

##### Statistical analysis

The statistical significance of ERP components will be tested by comparing their mean amplitudes to zero at ROIs using one-sample t-tests. This method will allow us to focus exclusively on significant responses, and to distinguish significant scalp distributions from noise. Statistically significant ERPs will be considered for further amplitude analysis.

For standard stimuli, levels of naturalness comparisons will be performed by Level × ROI repeated measures ANOVAs on each ERP and group independently. For deviant stimuli, Level × ROI × Deviant repeated measures ANOVAs will be performed.

Group comparisons will be conducted by Group × ROI repeated measures ANOVAs on each ERP and level of naturalness separately, for standard stimuli. Similarly, for deviant stimuli, Group × ROI × Deviant repeated measures ANOVAs will be performed. The effect of reducing the naturalness of voice on emotion processing will be assessed by the comparison between ERPs amplitudes at levels 1–5 for ASD group and ERPs amplitudes calculated in the control group for human voices. We expect to detect less natural voices to help typical processing in ASD children, that is, fewer difference with non-pathological processing of human voices at least natural voices. Based on MMN analysis at maximum peak ROI, the voice leading to the nearest to non-pathological processing in autistic children will be chosen for Stage 3.

Scalp distribution comparisons for standard stimuli will be assessed by Group × Laterality (FP1, F3, F7, T7, C3, P3, P7, O1/AFz, Fz, CPz, Cz, POz, Pz/FP2, F4, F8, T8, C4, P4, P8, O2) repeated measures ANOVAs on each ERP and level of naturalness separately. For deviant stimuli, Group × Laterality × Deviant repeated measures ANOVAs will be conducted.

For all amplitude analysis, normality will be assessed by Shapiro–Wilk test. In case of non-parametric distribution of the data, a transformation will be applied. Mauchly’s test of sphericity will be used to evaluate homogeneity of variances and co-variances. In case of violation of sphericity, a Greenhouse-Geisser correction will be applied in order to test main effects significance. *Post hoc* comparisons will be conducted to assess specific differences (Tukey and Dunnett procedures). In all cases, level of significance will be set at *p* < 0.05 and statistical analysis will be performed with R software.

#### Participants

Typically developed participants will be recruited from a public announcement posted on Tecnologico de Monterrey, Campus Monterrey. Autistic participants will be recruited by contacting parents of autistic children attending San Jose Hospital from Tecnologico de Monterrey.

Sample size estimation was calculated with G^∗^Power software version 3.1.9.7 (Faul et al., 2007) for Cohen’s d effect size index of 0.8, correlations among repeated measures of 0.5, statistical significance set at *p* < 0.05. For level comparisons, results indicated that 20 participants in each group would be enough to provide an estimated power of 0.90. For group comparisons, results indicated that 38 participants in each group would be enough to provide an estimated power of 0.91.

Forty high-functioning autistic volunteers aged 9 to 11-year-old will be involved in this stage of the research. All autistic participants will be diagnosed by a clinician according to DSM-V (Diagnostic and Statistical Manual of Mental Disorders, Fifth Edition) (American Psychiatric Association, 2013) or the International Classification of Diseases and Related Health Problems – Tenth Edition (World Health Organization, 1994) criteria. Autistic symptomatology will be assessed by the Autism Spectrum Rating Scales (ASRS) – parent report (Goldstein, 2012). A T-score greater than 60 (slightly elevated to very elevated scores for autistic behaviors) on Social/Communication, DSM-IV-TR (Text Revision), Peer and Adult Socialization and Social/Emotional Reciprocity domains will be considered as an inclusion criterion.

In addition, 40 TD volunteers aged 9 to 11-year-old will be included in this study. Similar to autistic children, ASRS will be used, and children with a score inferior to 60 in all scales will be included.

Any TD or autistic participant will be excluded if child:

• is under medication affecting the central or peripherical nervous system,
• has hearing loss or deficits, and/or
• has history of a developmental pathology and/or any disease affecting behavior and nervous system, apart from ASD for autistic participants.

### An Intervention Based on Social Stories^TM^, Synthesized Voices and Interactions With NAO

This last stage of the research aims to implement the use of the synthesized voice selected in Stage 2 in a 2-week intervention for autistic children. The intervention will aim to train emotions differentiation skills when triggered by prosody and will implement the Social Story^TM^ method while interacting with a social robot (NAO). Pre- and follow-up intervention assessments will be conducted using the ASRS questionnaire – parents form, completed with EEG and behavioral correlates of emotions differentiation. Considering the 4-week sensitivity of the ASRS questionnaire, mid- and end-intervention assessments will only be conducted by EEG and behavioral data analysis. A substantial improvement in social and emotional skills at end and follow-up assessments is expected.

#### Intervention

The intervention will consist of six-time per week sessions for 2 weeks conducted in a one-to-one format. Each session will last approximatively 10 min and will be composed of two Social Stories^TM^: one general and one specific. General Social Stories^TM^ will describe and compare all emotions. Specific ones will describe only one emotion. At the end of the first week, the child will have been presented with a specific story for each emotion and three different general stories. During the second week, the same stories will be repeated in a different order. Presentation order will be random and different for each participant. The stories will be uttered by NAO social robot that will be previously programmed using Choregraphe software (Femmam, 2017). Emotional and neutral speech will be uttered using the synthesized voiced selected in Stage 2. Each Social Story^TM^ will be written to train emotion differentiation and recognition through prosody (please see section “Social Stories^TM^ Elaboration” for more details and Supplementary File 1 for an example of a Social Story^TM^). Questions about the emotion conveyed will be asked to the participant. Correct responses will lead to a social reinforcement (e.g., “Well done!” ^∗^in Spanish^∗^ saying with a happy prosody and synthesized voice). Incorrect or absence of response will result in repeating the part of the story that contains the information necessary to answer the question. Then the question will be asked again, and a correct answer will lead to a social reinforcement. In case of incorrect answer, the correct answer will be told by the robot. In case of correct answers, the robot will specify that in daily life, this emotion may not be uttered by the synthesized voice but will be better uttered by his entourage with a more natural voice. Then the robot will repeat the same emotional utterance with the human voice and ask which emotion is conveyed. Again, correct responses will lead to social reinforcement. Incorrect or absence of response will result in repeating the part of the story that contains the information necessary to answer the question. Then the question will be asked again, and a correct answer will lead to a social reinforcement. In case of incorrect answer, the correct answer will be told by the robot. Each session will start with robot NAO friendly inviting the participant to play the “emotion game” and explaining the instructions. Robot’s movements and eye lighting will only be programmed for this part. During Social Stories^TM^, NAO will remain still in order to focus the child’s attention to the auditive information. All session long, NAO will be remotely controlled by the researcher with a Wizard-Of-Oz method using the Graphical User Interface provided by the system (Cao et al., 2019).

#### Outcome Measures

##### ASRS questionnaire

The ASRS questionnaire is a standardized, reliable and validated set of questions targeted to parents or teachers of autistic people aged between 2 and 18 years to measure their autistic symptomatology (Simek and Wahlberg, 2011). It presents several advantages for its use in research (Goldstein, 2012). First, a wide range of autistic social and emotional behaviors are assessed, so that it makes the evaluation of generalization of learning to other social domains possible. Second, the scores provided are based on a normative TD sample, thus it considers the autism spectrum as a continuum with the general population making possible the comparison with a TD control group. The full-length version for 6–18 years old children and youths is composed of 71 items divided in three scales: ASRS scale, DSM-IV, and Treatment Scale. A 4-level scale is presented to the rater in order him to specify how often a particular behavior is used to be observed in the child or youth that is evaluated. Higher scores indicate higher symptoms severity according to the rater. In this study, a particular focus will be dedicated to Social/Communication, DSM-IV-TR, Peer and Adult Socialization and Social/Emotional Reciprocity domains. Parents of both groups will answer this questionnaire on pre-intervention (1st session) and follow-up sessions (2 and 6 weeks after the end of the intervention).

##### Behavioral assessments

At pre- (1st session), mid- (7th session), end- (13rd session), and follow-up sessions (2 and 6 weeks after the end of the intervention), participants will be asked to answer the following behavioral task. It will consist of one block of stimulus pairs containing 244 pairs that will be presented at 60 dB via the Shure SRH1840 audio headset. The stimulus pair will be composed of either identical stimuli (50%; two neutral stimuli), or different stimuli [one neutral and one of the emotional deviants (50%; anger (10%), disgust (10%), fear (10%), happiness (10%), or sadness (10%)]. Fifty percent of pairs that contain a deviant stimulus will be composed of 1 neutral followed by 1 emotional stimulus, and 50% will be composed of 1 emotional followed by 1 neutral stimulus. Word pairs will be equally uttered with a male, woman, or child voice. The within-pair stimulus onset asynchrony will be 1,300 ms, and the between-pair stimulus onset asynchrony will be 2,600 ms. The order of stimulus pairs will be randomized between participants, but the stimulus sequence will be fixed. During this approximately 20-min task, participants will have to answer by pressing previously instructed keys on a laptop keyboard, if the emotional prosody of both stimuli of each pair will be the same or different. If they choose different, they will have to mention the emotion conveyed by the deviant (anger, disgust, fear, happiness, or sadness). Reaction times and correct/incorrect answers will be recorded. To make sure that the participant understands the task, three training trials will be presented before the test. Autistic participants will be accompanied by their parent if necessary.

##### EEG assessments

After behavioral assessment at pre- (1st session), mid- (7th session), end- (13rd session), and follow-up sessions (2 and 6 weeks after the end of the intervention), EEG data will be recorded while participants listen to a passive oddball paradigm. Emotional and neutral words uttered by human voices recorded at Stage 1 will be displayed to the participant. During the EEG recording, participants will sit on an armchair while watching a silent movie and will be instructed to pay no attention to the auditive stimuli. One stimulus block containing 1,200 words will be presented for an approximately 20-min paradigm. It will consist of 960 frequently presented neutral-uttered standard stimuli (80%), and occasional 48 anger (4%), 48 disgust (4%), 48 fear (4%), 48 happiness (4%), and 48 sadness (4%) deviant stimuli. Standard and deviant stimuli will be equally uttered with a male, woman, or child voice. The stimuli will be presented pseudo-randomly with deviant stimuli being preceded by at least two standard stimuli. The stimulus sequence will not vary between participants. The stimulus onset asynchrony will be of 1,300 ms. The stimuli will be presented at 60 dB via the Shure SRH1840 audio headset. Autistic participants will be accompanied by their parent if necessary.

Electroencephalographic recording and processing will follow the same methodology as the one described in section “EEG Recording and Processing.”

#### Social Stories^TM^ Elaboration

**FIGURE 2:**
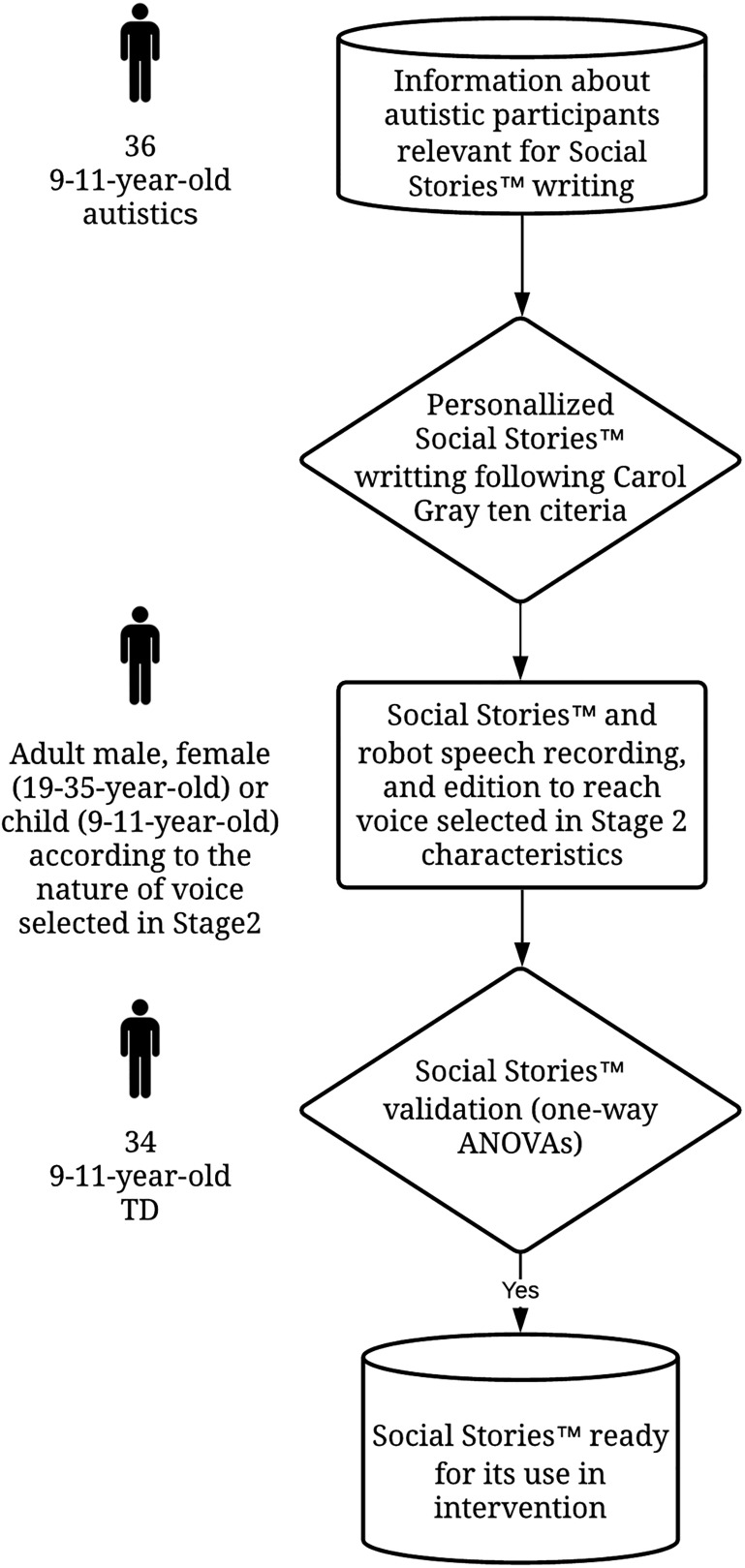
Process for Social Stories^TM^ elaboration before their use in intervention. TD, Typically developed.

Figure 2 illustrates the process that will be used to generate the Social Stories^TM^ before their use in the intervention.

##### Content

Social Stories^TM^ content will follow the ten criteria established by Gray (2015). Three general Social Stories^TM^ and six specific patterns will be initially written and will serve as basis for further child personalization. According to Carol Gray, each social story will contain three types of information: “The News,” “Ways to Think About The News,” and “Connections and Implications.” The News is an objective description of the social scenario that is trained. Ways to Think About The News is an indication of how managing The News adequately. Finally, Connections and Implications reference past, present, and future concrete examples of the social scenario that is trained in order to foster understanding and future correct behavior. Each Social Story^TM^ will be composed of an introduction, body part, and conclusion (Pane et al., 2015) in order to unsure a structured and logic design. Considering the age of the participants, Social Stories^TM^ will be made of at least 12 first and third-person perspective sentences that can be descriptive, perspective, coaching, affirmative, partial, or interrogative (Gray, 2015). To reach personalization to the child, relevant information about the child (e.g., interests, emotional usual behaviors) will be previously gathered by communicating with the parents. Then, three general and six specific Social Stories^TM^ will be written for each child. After writing them, they will be sent to the parents who will be asked if they wish to modify their content in order to foster the personalization to their child. Also, the child and parents will be free to add one or two sentences based on personal experience that would help to remember the information. Please see Supplementary Files 1, 2 for examples of a Social Story^TM^ and questions for the parents, respectively.

##### Recording and edition

Personalized and initial patterns of Social Stories^TM^, and all other robot speech will be recorded from volunteer actors: one adult male, one adult female, and one child, depending of the characteristics of the synthesized voices selected in Stage 2. They will be asked to read and get grips with the entire set of utterances and will be given as much time as he/she needs. Then he/she will be instructed to read each utterance with an adequate intonation. The emotional or neutral prosodies will be specified across the text. Participants will be asked to do their best possible to get into the emotional role. All specific utterances from a particular emotion will be uttered successively in order to facilitate this process. Participants will be asked to wait at least 5 s between two readings in order to focus before each recording. The recording material and environment will be the same than the one described in Stage 1.

Neutral and emotional synthesized speech uttered by the robot during the intervention will be obtained by editing acoustic features of the previous recordings in order to reach the same characteristics as neutral and emotional voices selected in Stage 2. The same methodology for synthesized voices creation as the one described in Stage 2 will be followed. The frequency response of the acoustic signal that comes from the robot’s speaker will be computed using Matlab. The frequency response will determine the deformation of the intensity of the sound by the speaker as a function of frequencies. Audio signals will be edited in order to compensate this bias generated by the speaker.

##### Validation

The previously created Social Stories^TM^ will be checked for adequacy regarding the understanding of emotions conveyed. TD children will take part to the validation task procedure. Participants will interact with NAO which will tell the Social Stories^TM^ with the adequate emotional prosody (please see Supplementary File 1 for an example of the prosody variations across a Social Story^TM^) uttered by the human voice from original recordings after the robot speakers frequency response compensated. For each story, the number of correct recognitions of emotions (correct/incorrect answers to the questions inserted in the story) will be counted. Before the beginning of the task, instruction will be explained. Particularly, it will be told to the participants that the stories are personalized for another child, so that they do not have to consider personalized sentences as directed to them. However, their task will be to answer the robot question about which emotion will be conveyed by the prosody. Correct responses will lead to social reinforcement. Each story and question will be told once to the participant. Each participant will listen to nine Social Stories^TM^ (patterns for three general and six specific stories) for an approximate 45-min session.

For number of correct/incorrect responses comparisons, a one-way ANOVA will be conducted for each Social Story^TM^ separately. Normality and homogeneity of variances will be assessed by Shapiro–Wilk and Bartlett tests, respectively. In case of non-parametric distribution of the data, Kruskal–Wallis test will be applied. *Post hoc* comparisons will be conducted to assess specific differences (Tukey after ANOVA, Wilcoxon tests with *p*-value adjustment by Holm method after Kruskal–Wallis). Level of significance will be set at *p* < 0.05. If a Social Story^TM^ does not reach significance, it will be modified and validated again.

#### Data Analysis

##### Statistical analysis on ASRS scores

Group comparisons will be conducted by one-way ANOVAs on T-scores for each assessment session and symptom domain (Social/Communication, DSM-IV-TR, Peer and Adult Socialization, and Social/Emotional Reciprocity) separately.

Time comparisons (between assessment sessions) will be tested by one-way ANOVAs on T-scores for each group and symptom domain (Social/Communication, DSM-IV-TR, Peer and Adult Socialization and Social/Emotional Reciprocity) separately.

Normality and homogeneity of variances will be assessed by Shapiro-Wilk and Bartlett tests, respectively. In case of non-parametric distribution of the data, Kruskal-Wallis test will be applied. Post-hoc comparisons will be conducted to assess specific differences (Tukey procedure in case of ANOVA, Wilcoxon tests with *p*-value adjustment by Holm method in case of Kruskal–Wallis test).

##### Statistical analysis on behavioral data

Group comparisons of reaction times will be conducted by a Group × Stimulus (neutral, anger, disgust, fear, happiness, or sadness) repeated measures ANOVAs on each assessment session separately. Time comparisons will be conducted by Assessment Session × Stimulus on each group separately. Normality of reaction times will be assessed by Shapiro–Wilk test. In case of non-parametric distribution of the data, a transformation will be applied. Mauchly’s test of sphericity will be used to evaluate homogeneity of variances and co-variances. In case of violation of sphericity, a Greenhouse-Geisser correction will be applied in order to test main effects significance. Post-hoc comparisons will be conducted to assess specific differences (Tukey procedure).

Group comparisons of number of correct answers will be conducted by one-way ANOVAs for each deviant and assessment session separately. Time comparisons (between assessment sessions) will be tested by one-way ANOVAs on number of correct answers for each deviant and each group separately. Normality and homogeneity of variances will be assessed by Shapiro–Wilk and Bartlett tests, respectively. In case of non-parametric distribution of the data, Kruskal–Wallis test will be applied. *Post hoc* comparisons will be conducted to assess specific differences (Tukey after ANOVA, Wilcoxon tests with *p*-value adjustment by Holm method after Kruskal–Wallis).

##### ERPs

For ERP amplitude analysis, electrodes will be grouped according to ROIs described in section “Data Analysis.” Maximum peak time windows used for data analysis of ERPs will be identical to the one mentioned in section “Data Analysis.” MMN, P200, P3a, LPP will be under study.

Event-Related Potentials will be extracted by averaging the data obtained for standard and deviant stimuli, and for each condition independently. The ERPs elicited by standard stimuli will be subtracted from the ERPs elicited by deviants to calculate difference waves. The mean ERP amplitude will be calculated as follows. For standard stimuli, group and assessment session average peak latencies will be determined from all ROIs for TD and ASD separately. Mean amplitudes will be determined by considering a ±50-ms time window centered to the grand mean peak latency. For deviant stimuli, mean amplitudes will be calculated from the difference waves at ROIs. Mean amplitudes will be determined by considering a ±50-ms time window centered to the grand mean peak latency (Kujala et al., 2007; Paris et al., 2018).

##### Statistical analysis on ERPs data

The statistical significance of ERP components will be tested by comparing their mean amplitudes to zero at ROIs using one-sample *t*-tests. This method will allow us to focus exclusively on significant responses, and to distinguish significant scalp distributions from noise. Statistically significant ERPs will be considered for further amplitude analysis.

Group comparisons will be conducted by Group × ROI repeated measures ANOVAs on each ERP and assessment session separately, for standard stimuli. Similarly, for deviant stimuli, Group × ROI × Deviant repeated measures ANOVAs will be performed.

For standard stimuli, time comparisons will be conducted by Assessment Session × ROI repeated measures ANOVAs on each ERP and group separately. For deviant stimuli, Assessment Session × ROI × Deviant repeated measures ANOVAs will be performed.

Scalp distribution comparisons for standard stimuli will be assessed by Group × Laterality (FP1, F3, F7, T7, C3, P3, P7, O1/AFz, Fz, CPz, Cz, POz, Pz/FP2, F4, F8, T8, C4, P4, P8, O2) repeated measures ANOVAs on each ERP and assessment session separately. For deviant stimuli, Group × Laterality × Deviant repeated measures ANOVAs will be conducted.

For all amplitude analysis, normality will be assessed by Shapiro–Wilk test. In case of non-parametric distribution of the data, a transformation will be applied. Mauchly’s test of sphericity will be used to evaluate homogeneity of variances and co-variances. In case of violation of sphericity, a Greenhouse-Geisser correction will be applied in order to test main effects significance. *Post hoc* comparisons will be conducted to assess specific differences (Tukey procedure).

All statistical analysis will be performed with R software. Level of significance will be set at *p* < 0.05.

#### Participants

Typically developed participants will be recruited from a public announcement posted on Tecnologico de Monterrey, Campus Monterrey. ASD participants will be recruited by contacting parents of autistic children attending San Jose Hospital from Tecnologico de Monterrey.

##### Social Stories^TM^ and robot utterances recordings

Participants will be healthy TD volunteers. Adults will be aged 19–35 and children will be aged 9–11 years old. One participant for each type of voice will be included (e.g., one adult male). The same inclusion and exclusion criteria than the one described in Stage 1 will be followed.

##### Validation of Social Stories^TM^

Sample size estimation was calculated with G^∗^Power software version 3.1.9.7 (Faul et al., 2007) for Cohen’s d effect size index of 0.8, statistical significance set at *p* < 0.05. Results indicated that 34 participants would be enough to provide an estimated power of 0.90. Therefore, 40 healthy TD aged between 9 and 11 years old will volunteer. Participants will be excluded if they have a clinical history of any pathology that affects emotional behavior, hearing, or speech at the time of the study.

##### Intervention

Autistic and TD participants involved in this study will have to meet the same criteria than those described in Stage 2. However, participants from Stage 2 will not be included in Stage 3 in order to avoid any bias elicited by a former exposition to the synthetic voices under study.

Sample size estimation was calculated with G^∗^Power software version 3.1.9.7 (Faul et al., 2007) for Cohen’s d effect size index of 0.8, correlations among repeated measures of 0.5, and statistical significance set at *p* < 0.05. For ERP analysis, results indicated that 13 participants in each group would be enough to provide an estimated power of 0.92 for group comparisons, and 11 participants in each group would be enough to provide an estimated power of 0.91 for assessment sessions comparisons. For behavioral analysis on reaction times, results indicated that 13 participants in each group would be enough to provide an estimated power of 0.91 and seven participants in each group would be enough to provide an estimated power of 0.93 for assessment sessions comparisons. For behavioral analysis on number of correct/incorrect answers, results indicated that 18 participants in each group would be enough to provide an estimated power of 0.92 for group comparisons, and 15 participants in each group would be enough to provide an estimated power of 0.90 for assessment sessions comparisons (Cohen’s d effect size index of 0.8, statistical significance set at *p* < 0.05). For behavioral analysis on ASRS scores, results indicated that 16 participants in each group would be enough to provide an estimated power of 0.92 and 18 participants in each group would be enough to provide an estimated power of 0.90 for assessment sessions comparisons.

A total of 72 participants will take part to this Stage and will be divided in four groups:

• Group 1: autistic children receiving robot, synthesized and human voices intervention (18 participants).
• Group 2: autistic children receiving robot and human voice intervention (18 participants).
• Group 3: autistic children without receiving intervention (18 participants).
• Group 4: TD children without receiving intervention (18 participants).

Group 1 will be the experimental group and groups 2–4 will be controls. Children from group 2 will receive the same intervention as children from group 1, with the exception of the use of human voices only. This group will help to evaluate the efficacy of the use of synthesized voices in the intervention. Autistic participants will be randomly assigned to any of groups 1, 2 or 3. All four groups will be included in pre-, mid-, end and follow-up assessments.

## Discussion

This project of investigation aims to fulfill three main gaps of the current scientific literature. First, acoustic parameters from Castilian and Mexican Spanish will be analyzed in order to validate a newly formed emotion speech database for Mexican Spanish and to highlight cross-cultural differences in the way of expressing emotions by prosody. The first issue was to determine which acoustic features should be considered to optimize the distinction from one emotion to another. As no reliable set of acoustic features exists yet, a literature review of prosodic emotional speech parameters was conducted. MFCC coefficients were the most usually extracted features for machine learning applications. This analysis consists of computing the log values of a linear cosine transform of spectral parameters on a Mel scale, thus considering the human particularities of frequency responses (Caballero-Morales, 2013). Other recurrent spectral features were the pitch (fundamental frequency), formants, jitter, shimmer and harmonics-to-noise ratio (Swain et al., 2018). Formants are high-energy frequencies acoustic resonances of the vocal tract (Gelfand, 2009). They are generally labeled F1, F2, F3, and so on, from the lowest formant to the highest: the first three- formants are usually extracted for emotions discrimination (Kamińska, 2019). Jitter and shimmer refer to a period-to-period fluctuation of the pitch and the energy, respectively (Arruti et al., 2014). The harmonics-to-noise ratio considers the energy of the harmonics according to the one of other frequencies. It is directly correlated to the voice quality according to breathiness and roughness and is a principal component to characterize human voices (Arruti et al., 2014). The energy, intensity and rate were other features commonly used for emotional prosody recognition (Swain et al., 2018). Therefore, it is suitable to extract those acoustic parameters for the present work and predict high accuracy in emotions discrimination and classification. However, previous studies have also highlighted the influence of linguistic parameters on emotions processing (Méndez-Bértolo et al., 2011; Palazova et al., 2011; Hinojosa et al., 2014). Therefore, it is necessary to supervise linguistic values, for all stimuli to be nouns and adjectives, valence, arousal, and emotional category to be in accordance with prosody, and frequency, concreteness, and familiarity matched between emotions, so that the unbiased allocation of prosody during recordings may be ensured. Controlling for these factors will add reliability to our database and enrich the current available material, as previous works for Spanish emotional utterances did not systematically control for linguistic features (Caballero-Morales, 2013; Hinojosa et al., 2016a, b; Pérez-Espinosa et al., 2020).

The second aim of the present work is to determine the essence of the naturalness of human voices compared to synthesized ones by analyzing acoustic features. Previous studies have highlighted the human specific ability to recognize the human shape of human voices over synthesized ones (Tamura et al., 2015; Kuriki et al., 2016). For instance, Tamura et al. (2015) emphasized that human voices are generally perceived as more natural (as compared to awkward), human (as compared to robotic), animated, familiar, comfortable (as compared to irritating), emotional and warmer than artificial voices. Furthermore, the left posterior insula was outlined as a marker of the perception of the ecological validity with greater activation when processing human versus artificial voices. Therefore, by their highly natural identity, human voices may have a particular acoustic profile that allow their conspecific- and socially related neuronal processing and perception. To the best of our knowledge, the present work will be the first to detangle the acoustic identity of the human naturalness in voices compared to synthesized ones. Autistics have demonstrated impairments to associate human versus artificial voices with familiarity, warmth, emotions, animateness, naturalness (as compared to unnatural), and humanness (as compared to mechanics), and this atypical perception was correlated to the general autistic symptomatology (Kuriki et al., 2016). Therefore, ASD are characterized by impairments in the ability to discern social and human-related cues in human voices compared to synthesized ones, which invalidate them for further socio-emotional interactions. The present work proposes to reduce the human and natural-related acoustic information in voiced emotional utterances in order to create a more mechanistic and stereotyped environment which would be more comfortable and increase attention and interest to emotional stimuli in autistics. By gradually removing naturalness in voice, we pretend to identify up to which grade this process may help to improve emotional prosody discrimination in autistics. As a secondary outcome, it would be characterized how emotional processing preferences may vary according to human-likeness (Mori et al., 2012).

Finally, the present project of investigation seeks to explore the EEG event-related basis of emotional prosody uttered by human and synthesized voices processing in autism. Also, the emotions processing-related social robot-induced neuronal plasticity will be analyzed. Previous studies outlined significant plastic neuronal changes correlated to behavioral improvements after drug-free interventions in autistics, even in patients older than early critical plasticity periods. Particularly, Van Hecke et al. (2015) observed a significant alignment to typical neuronal resting activity highlighted by a shift from right to left hemisphere dominance of gamma oscillation activity after 14 sessions of the Program for the Education and Enrichment of Relational Skills in autistic adolescents (mean age = 14-year-old) which correlated to social abilities improvements. Using and ERP analysis before and after training for face processing, Faja et al. (2012) emphasized a post-training reduction of the amplitude of the P1 component in autistic adults (mean age = 22-year-old) stressing a potentially lower early attentional effort for face processing, in line with behavioral improvements. Nevertheless, no social robot-based intervention assessed pre- and post-electrophysiological correlates yet. Therefore, the present study suggests fulfilling this gap by highlighting the neuronal plasticity associated with the effects of a social robot-based intervention directed toward emotional prosodies discrimination skills. Behavioral improvements as measured by the ASRS questionnaire and responses to the experimental task are anticipated. Measuring behavioral outcomes by the validated ASRS questionnaire increases this work’s reliability and allows to extend results to daily life and human interactions as well as to improvements beyond (yet related to) the primary-trained skill (Yun et al., 2017; So et al., 2018; David et al., 2020). The enhancement of the intervention by synthesized voices and a social robot may lead to a multisensorial, real-life-like, safe, controllable, and simplified environment that may foster positive outcomes.

This project of investigation might encounter some pitfalls and unexpected results. Non-professionalism of actors recruited for emotional recordings during Stage 1 may affect the reliability of required prosodies. Nevertheless, the number of volunteers will be increased in order to generate four times more utterances than necessary to enable the selection of best actor participants. Furthermore, emotion recognition accuracy will be validated by supervised learning algorithm to ensure the quality of emotion discrimination based on acoustic features. Stage 2 aims to explore emotional recognition impairments in autism triggered by naturalness of human voices. We expect to observe a diminution of the amplitude of ERPs in the autistic population as a reflect of decreased attentional, sensorial, and cognitive emotional processing (Lartseva et al., 2015; Lindström et al., 2016, 2018). The autistic condition may also be characterized by hemispheric lateralization anomalies due to lateralized compensatory brain activity (Subbaraju et al., 2018). Impairments are expected to fade with the decreasing naturalness of voice stimuli. However, in the opposite case, no effect of naturalness on neural emotional processing by autistic children would be an evidence that the modulated acoustic parameters would not participate to autistic impairments. In the latter situation, or in case of enhancement of autistic impairments triggered by naturalness modulations, the acoustic edition of the stimuli will be reconsidered. Finally, Stage 3 expects the implementation of less natural voices in a robot-based intervention with the aim of improving emotion detection by autistic children. The 2-week intervention may imply a high rate of withdrawals whereas the sample size needed is relatively high. However, upon completion, we expect the intervention to trigger a reduction of autistic impairments (reduction of symptomatology, higher EEG activity, higher behavioral accuracy for emotion discrimination: smaller reaction times, and higher correct answers at end- and follow-up assessments).

## Conclusion

The present research project aims to contribute to the current scientific knowledge by expecting to fulfil the following novel goals: (1) an emotional speech database adapted to the Mexican cultural shape of prosodic expressions will be created, (2) naturalness in voice will be defined and evidence that it triggers difficulty to discriminate emotional prosodies in ASD will be empirically supported, (3) a Social Stories^TM^ and robot-based intervention will be implemented to test the efficacy of using less-natural voices to help typical emotional discrimination in autistic children.

## Ethics Statement

This protocol has been registered at BioMed Central under the following number: ISRCTN18117434. The studies involving human participants were reviewed and approved by Ethical Committee of the School of Medicine of Tecnologico de Monterrey (register number within the National Committee of Bioethics CONBIOETICA 19 CEI 011-2016-10-17) under the following number: P000409-autismoEEG2020-CEIC-CR002 on July 14th, 2020. Written informed consent to participate in this study will be provided by all participants and the participants’ legal guardian/next of kin in case of children.

## Author Contributions

MD wrote the manuscript. All authors contributed to the conception and design of the protocol, manuscript revision, and read and approved the submitted version.

## Conflict of Interest

The authors declare that the research was conducted in the absence of any commercial or financial relationships that could be construed as a potential conflict of interest.

## Supplementary Material

Supplementary File 1 Example of a general Social Story^TM^.

Supplementary File 2 Example of questions directed to children’s parents to help personalizing Social Stories^TM^.
