# Supplementary material for: Electroencephalographic Correlate of Mexican Spanish Emotional Speech Processing in Autism Spectrum Disorder: To a Social Story and Robot-Based Intervention
Source: Front Hum Neurosci. 2021 Feb 26;15:626146. doi: 10.3389/fnhum.2021.626146 (PMC7952538; doi:10.3389/fnhum.2021.626146)
Supplement: Supplementary File 1 — Example of a general Social StoryTM. [file Table_1.docx]

**Example of a general Social Story^TM^.**

*Context:* John is a 9-year-old autistic child. He loves his dog and often asks to play with him in the garden. He has a recent obsession for collecting stamps. He hates spinaches and is afraid of dark.

*Title/Introduction:* We all express emotions in our voice.

*Body part:* People *(who)* often feel emotions *(affirmative sentence).* They can feel sadness (*said with a sad prosody)*, anger (*said with an angry prosody)*, fear (*said with an afraid prosody)*, disgust (*said with a disgusted prosody)*, happiness (*said with an happy prosody)*, or they can just do not feel any particular emotion and be in a neutral state *(said with an neutral prosody)*. For instance, people used to feel sad when their dog dies *(when, why, when; descriptive sentence)*. Adults can feel angry if I do not respect the school’s rules (*descriptive sentence)*. I feel scared when I am alone in a dark room *(perspective sentence)*. People use to be disgusted when they eat something they do not like (*descriptive sentence).* When someone is happy, generally other people around him or her is happy too *(descriptive sentence).* People use to be in a neutral state when nothing is changing their usual state *(descriptive sentence).* I can know what someone is feeling by focusing in his or her voice *(coaching sentence).* I can think of my mum saying, “Happy birthday” *(said with a happy voice)* to me to recognize happiness *(self-coaching sentence).* Speech rate and volume can also help a lot to know which emotion is conveyed *affirmative sentence)*. When someone is sad, he or she may speak slowly and quietly (*descriptive sentence).* I am used to speak like this when I lose my favorite stamp *(self-coaching sentence).* Neglect *(said with a sad prosody)*. What is the emotion here? When people are angry, they use to speak loudly and slowly *(descriptive sentence).* Abuse *(said with an angry prosody).* What is the emotion here? When someone is afraid, he or her may have a rapid and loudly speech (*descriptive sentence)*. Fright *(said with an afraid prosody).* What is the emotion here? When people are disgusted, they use to speak slowly (*descriptive sentence)*. I usually speak like that when I have to eat spinaches *(self-coaching sentence)*. Slugs *(said with a disgusted prosody).* What is the emotion here? When someone is happy, his or her speech may be loud and quick *(descriptive sentence).* Hug *(said with a happy prosody).* What is the emotion here? Neutral state can be characterized by a medium rate and volume *(affirmative sentence)*. Farmer *(said with a neutral voice).* What is the emotion here?

*Conclusion:* We all can feel emotions *(affirmative sentence)*. People can feel sad, angry, afraid, disgusted, happy, or neutral *(descriptive sentence).* I can recognize emotions paying attention to the voice *(coaching sentence).*

*Following Social Story Formula:* $\frac{Number of sentences that describes}{Number of coach sentences}= \frac{16}{5} \geq2$
