# Supplementary material for: Electroencephalographic Correlate of Mexican Spanish Emotional Speech Processing in Autism Spectrum Disorder: To a Social Story and Robot-Based Intervention
Source: Front Hum Neurosci. 2021 Feb 26;15:626146. doi: 10.3389/fnhum.2021.626146 (PMC7952538; doi:10.3389/fnhum.2021.626146)
Supplement: Supplementary File 2 — Example of questions directed to children’s parents to help personalizing Social StoriesTM. [file Table_2.docx]

**Example of questions directed to children’s parents to help personalizing Social Stories^TM^.**

What are your child current favorite interests?

What does your child do not like at all (activities, food, environments, for instance)?

Does he/she have a recent obsession? (collectable pins, cards, stamps, specific type of music, dress style, for example).

What generally makes your child happy? Sad? Angry? Afraid? Disgusted?

Can you remember particular contexts in which your child could understand someone’s emotional state? (his/her friend/sister/brother/cousin/classmate being sad/happy/angry/feared/disgusted, for instance).

**After writing the Social Stories^TM^, they will be sent to the parents to have their prior approval and feedback.**

Does your child or do you wish to add a sentence that could help to remember the information in the story (self-coaching sentences)?
